# Identification of five genetic variants with differential effects on obesity-related traits based on age

**DOI:** 10.3389/fgene.2022.970657

**Published:** 2022-10-07

**Authors:** Ju Yeon Chung, Hae-Un Jung, Dong Jun Kim, Eun Ju Baek, Han Kyul Kim, Ji-One Kang, Ji Eun Lim, Bermseok Oh

**Affiliations:** ^1^ Department of Biomedical Science, Graduate School, Kyung Hee University, Seoul, Korea; ^2^ Department of Biochemistry and Molecular Biology, School of Medicine, Kyung Hee University, Seoul, Korea

**Keywords:** obesity-related trait, genetic variant, age, stratified analysis, genome-wide association study

## Abstract

Obesity is a major public health concern, and its prevalence generally increases with age. As the number of elderly people is increasing in the aging population, the age-dependent increase in obesity has raised interest in the underlying mechanism. To understand the genetic basis of age-related increase in obesity, we identified genetic variants showing age-dependent differential effects on obesity. We conducted stratified analyses between young and old groups using genome-wide association studies of 355,335 United Kingom Biobank participants for five obesity-related phenotypes, including body mass index, body fat percentage, waist-hip ratio, waist circumference, and hip circumference. Using *t*-statistic, we identified five significant lead single nucleotide polymorphisms: rs2258461 with body mass index, rs9861311 and rs429358 with body fat percentage, rs2870099 with waist-hip ratio, and rs145500243 with waist circumference. Among these single nucleotide polymorphisms, rs429358, located in *APOE* gene was associated with diverse age-related diseases, such as Alzheimer’s disease, coronary artery disease, age-related degenerative macular diseases, and cognitive decline. The C allele of rs429358 gradually decreases body fat percentage as one grows older in the range of 40–69 years. In conclusion, we identified five genetic variants with differential effects on obesity-related phenotypes based on age using a stratified analysis between young and old groups, which may help to elucidate the mechanisms by which age influences the development of obesity.

## Introduction

Obesity is a major risk factor for complex diseases such as cardiovascular diseases and metabolic syndrome (Grundy, 2000) and has become a worldwide public health problem as its prevalence is increasing in most developed countries (OECD, 2017). As life expectancy has increased, the prevalence of obesity has also steadily increased among older people (Jura and Kozak, 2016). This increased prevalence is related to changes in body composition, such as an increase in fat mass and decline in lean mass (Slawik and Vidal-Puig, 2006; Miard and Picard, 2008; Barzilai et al., 2012; Zamboni and Mazzali, 2012). Additionally, retirement of old people may create a change in their lifestyle that causes a positive energy balance state leading to excess fat tissue accumulation (Tchkonia et al., 2010).

Over the past 20 years, genome-wide association studies (GWASs) have been conducted on obesity-related traits. Yengo et al. (2018) identified 941 independent single nucleotide polymorphisms (SNPs) associated with body mass index (BMI) using 681,275 European ancestry samples from both the United Kingom biobank (UKB) and genetic investigation of anthropometric traits (GIANT) Consortium. Similarly, Pulit et al. (2019) identified 463 waist-hip ratio (WHR)-associated SNPs using 694,649 samples from UKB and GIANT. Further studies have reported that the genome-wide SNP-heritability (*h*_snp_^2^) for BMI and WHR was 28% and 17%, respectively (Hou et al., 2019). Additionally, a few studies have investigated whether genetic effects differ between old and young individuals.

Winkler et al. studied genetic variants with different age-dependent genetic effects on BMI and WHR using the GIANT (Winkler et al., 2015) and found 15 loci with statistical significance (at 5% FDR) for BMI, although they did not find any loci for WHR. Robinson et al. found that the gene–age interaction explained 8.1% of the BMI variance when they performed genome-wide interaction analysis between genetic variants and age (Robinson et al., 2017). Ge et al. investigated the heritability of BMI as the age of a group changes and found that BMI heritability significantly decreases with age (Ge et al., 2017). These results suggest that age influences the genetic effects on obesity-related traits. Genetic components that show different effects depending on age could be useful to provide specific pathways affecting obesity in young and old age groups. That is, by understanding which genes are affected by age, metabolic pathways can be targeted more specifically for future treatments (Choh et al., 2014). However, few studies have identified the genetic variants that interact with age for differential effects on obesity.

In this study, we performed stratified analyses using GWASs of five obesity-related phenotypes by grouping samples by age using 355,335 unrelated European descendants of the UKB to identify genetic variants with differential effects between young and old individuals.

## Materials and methods

### Study population

The UKB is a population-based cohort that recruited 502,620 individuals aged 37–73 years in the United Kingdom during 2006–2010 (Collins, 2012). We excluded samples based on the following criteria provided by the Neale lab (http://www.nealelab.is/uk-biobank): 1) related samples that were excluded from principal component (PC) analysis, 2) sex chromosome aneuploidy, 3) non-European descendants estimated by PCs, and 4) non-White British samples based on self-reported ethnic background (white British, Irish, and white). After quality control of the samples, 355,335 individuals were selected for further analyses.

### Phenotypic data

We selected five obesity-related phenotypes including BMI, body fat percentage (BFP), WHR, waist circumference (WC), and hip circumference (HC). BMI was calculated as weight (field ID: 21,002) divided by square of height (field ID: 12,144) (kg/m^2^). WC (field ID: 48) and HC (field ID: 49) were selected, and WHR was calculated as WC divided by HC. BFP (Field ID: 23,099) was estimated using impedance measurements. Additionally, age at recruitment (field ID: 21,022), sex (field ID: 31), genotyping array (field ID: 22,000), PCs (field ID: 22,009), and TDI (field ID: 189) were selected as covariates for GWASs. And LDL (field ID: 30,780), and HDL (field ID: 30,760) were selected for testing the association with rs429358. All phenotypic data were from baseline (at recruitment).

### Genotypic data

The UKB genotyped 487,409 participants using the UKB Axiom array and the United Kingom BiLEVE Axiom array from Affymetrix (Santa Clara, CA, United States) (Sudlow et al., 2015; Bycroft et al., 2018). Genotyping was performed using the United Kingom10 K Project and 1,000 Genome Project Phase 3 reference panels (Huang et al., 2015). SNP quality control procedures were applied to 93,095 623 imputed SNPs based on the following exclusion criteria: SNPs with missing genotype call rates >0.05, minor allele frequency <0.01, and *p*-value for Hardy-Weinberg equilibrium test <1.00 × 10^–6^. In total, 5,664 578 SNPs were retained for further analysis.

### Statistical analysis

Because we wanted to compare each age group with the same sample size, we divided the subjects into quartiles based on age at recruitment (Q1, Q2, Q3, and Q4). And we performed analyses of genome-wide association studies (GWAS) between genetic variants and five obesity-related phenotypes separately, using the linear regression model adjusted for age, sex, genotyping array, and PC1–10 in each age group with PLINK v.1.90 software (Purcell et al., 2007).

To analyze the age-stratified effect of SNPs, first we estimated the correlation among age groups. The correlation coefficient, *r*, was calculated using the Spearman rank across all SNPs of Q1, Q2, Q3, and Q4. Second, we selected the pair of age groups with the lowest r in each obesity-related trait. And then we computed differential *p*-values (*P*_diff_) by testing the difference in beta coefficients between age groups using *t*-statistic as follows (Winkler et al., 2015):

$$t=\frac{b_{1}-b_{2}}{\sqrt{{SE}_{1}^{2}+{SE}_{2}^{2}-2r∙{SE}_{1}∙{SE}_{2}}}$$

$b_{1}$ and ${SE}_{1}$ , and $b_{2}$ and ${SE}_{2}$ are the beta coefficients and standard errors for each SNP in the age group 1 and 2, respectively. *r* is the correlation coefficient between the beta coefficients of age group 1 and 2. Stratified analysis and correlation analysis were performed using R v3.6.0 software. The GWAS significance threshold (*P*_diff_ < 5 × 10^−8^) was considered statistically significant to account for multiple testing. The lead SNPs were identified by FUMA (https://fuma.ctglab.nl), an online platform that provides functional annotation, visualization and interpretation of GWAS results. We carried out clumping using FUMA to identify lead SNPs based on the clumping conditions as follows; *P*_diff_ < 5 × 10^–8^, *r* ^2^ < 0.1, and distance between LD blocks >250 kb. The linkage disequilibrium (LD) information, *r* ^2^, was computed using 1,000 Genome Project phase 3 European as a reference panel.

Manhattan plots and QQ plots were generated using R v3.6.0 software. Regional plots for significant loci were created using LocusZoom (Pruim et al., 2010) and linkage disequilibrium (LD) was presented based on a European sample from the 1,000 Genome Project phase I reference panel (1000 Genomes Project Consortium et al., 2010).

### Functional annotation tools

To analyze the biological functions of age-stratified SNPs, we used several approaches, including HaploReg V4.1 (Ward and Kellis, 2012), GTEx V8 (Consortium, 2020), GWAS Catalog (Buniello et al., 2019), and PhenoScanner V2 (Kamat et al., 2019). HaploReg was used to search for the effect of the identified SNPs on the transcription factor-binding site motif and perform enhancer enrichment analysis. The GTEx database was used to evaluate the association between genetic variations and gene expression. The GWAS Catalog and PhenoScanner databases were further used to search for associations between genetic variants and a broad range of phenotypes.

## Results

**FIGURE 1:**
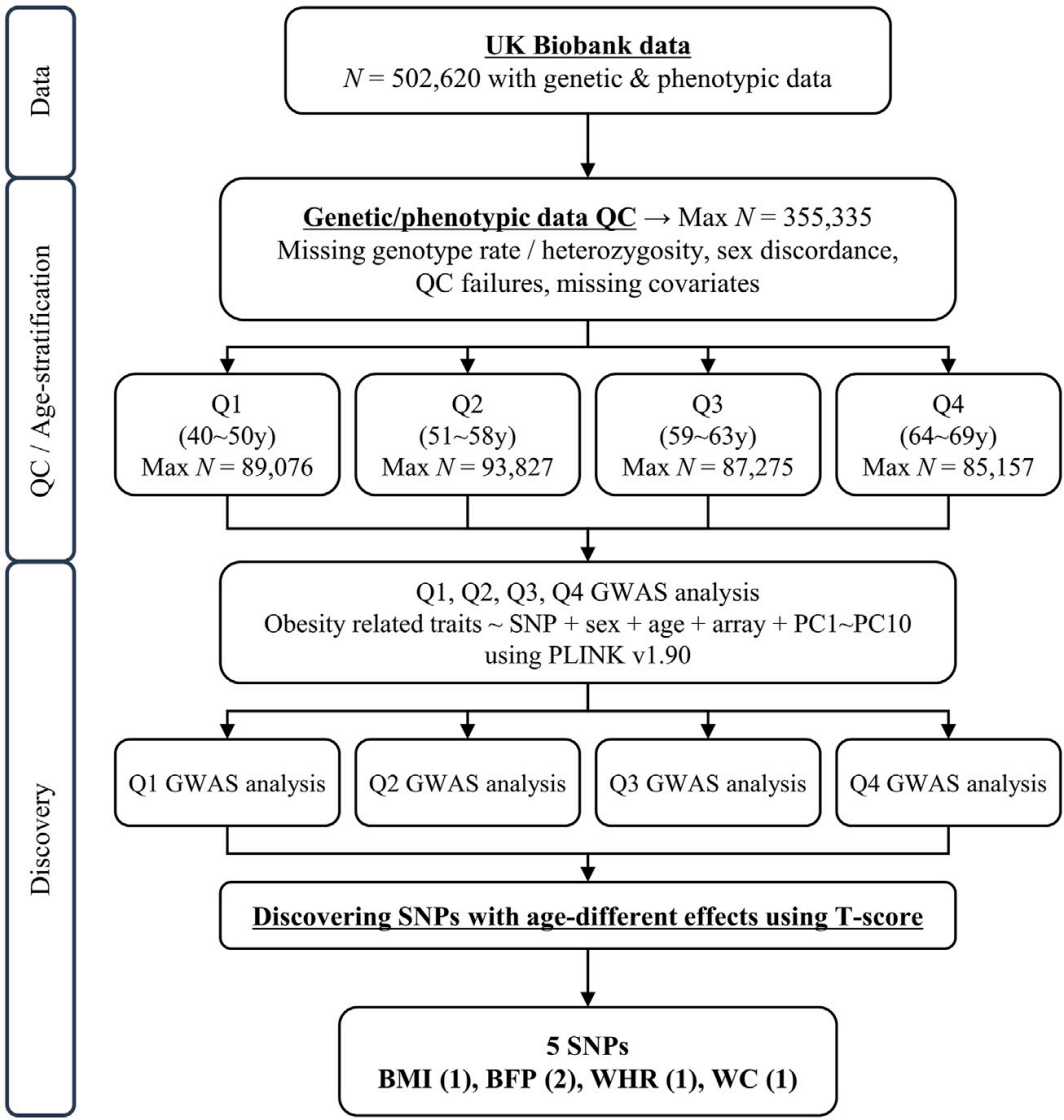
Schematic diagram of study design. N, sample size; QC, quality control; GWAS, genome-wide association study; UKB, United Kingom Biobank.

After the quality control of samples described in the Methods section, the sample size used in this study consisted of 355,335 individuals (40–69 years) from White British and Irish populations in the UKB. The 355,335 individuals were divided into quartiles, with a similar number of individuals in each group for further analysis of GWAS (Figure 1). The basic characteristics of these groups are presented in Table 1 (Q1: 40–50 years, Q2: 51–58 years, Q3: 59–63 years, and Q4: 64–69 years). The mean age of each quartile group is 45.47 years, 54.68 years, 61.07 years, and 66.28 years in Q1, Q2, Q3, and Q4, respectively. And the age range in each group is 10, 7, 4, and 5 years in Q1, Q2, Q3, and Q4, respectively. All groups had a lower percentage of men than women, in the range 43.93%–49.47%. The mean values for obesity-related phenotypes, such as BMI, BFP, WHR, WC, and HC, in each group gradually increased with age, although those of Q4 were similar to those of Q3. These trends are presented in Supplementary Figure S1. In addition, the association between age and these phenotypes was analyzed by linear regression adjusted for sex, and all phenotypes showed statistical significance in a positive direction (Supplementary Table S1).

**TABLE 1:** Basic characteristics of the United Kingom Biobank cohort.

Phenotype		Q1	Q2	Q3	Q4
BMI (kg/m^2^)	N (%)	88,977 (45.52%)	93,693 (44.02%)	87,111 (45.76%)	85,009 (49.63%)
	age	45.57 ± 3.02	54.69 ± 2.30	61.08 ± 1.36	66.28 ± 1.69
	BMI	26.97 ± 4.92	27.46 ± 4.96	27.57 ± 4.68	27.53 ± 4.39
BFP (%)	N (%)	87,887 (45.55%)	92,541 (43.93%)	85,767 (45.66%)	83,345 (49.47%)
	age	45.57 ± 3.01	54.68 ± 2.30	61.07 ± 1.36	66.28 ± 1.69
	BFP	29.72 ± 8.63	31.52 ± 8.58	32.04 ± 8.44	32.09 ± 8.18
WHR	N (%)	89,060 (45.54%)	93,815 (44.03%)	87,258 (45.77%)	85,152 (49.64%)
	age	45.57 ± 3.01	54.69 ± 2.30	61.08 ± 1.36	66.28 ± 1.69
	WHR	0.85 ± 0.09	0.87 ± 0.09	0.88 ± 0.09	0.89 ± 0.09
WC (cm)	N (%)	89,076 (45.55%)	93,827 (44.03%)	87,275 (45.78%)	85,157 (49.64%)
	age	45.57 ± 3.02	54.69 ± 2.30	61.08 ± 1.36	66.28 ± 1.69
	WC	88.10 ± 13.52	90.03 ± 13.80	91.13 ± 13.33	92.01 ± 12.89
HC (cm)	N (%)	89,062 (45.54%)	93,817 (44.03%)	87,258 (45.78%)	85,156 (49.64%)
	age	45.57 ± 3.02	54.69 ± 2.30	61.08 ± 1.36	66.28 ± 1.69
	HC	103.06 ± 9.28	103.61 ± 9.48	103.60 ± 9.06	103.38 ± 8.64

Values are presented as total number (N) and male percentage (%) in each group, or mean ± standard deviation (SD).

### Stratified analysis of genome-wide association studies identified age-dependent genetic variants for obesity-related phenotypes.

**FIGURE 2:**
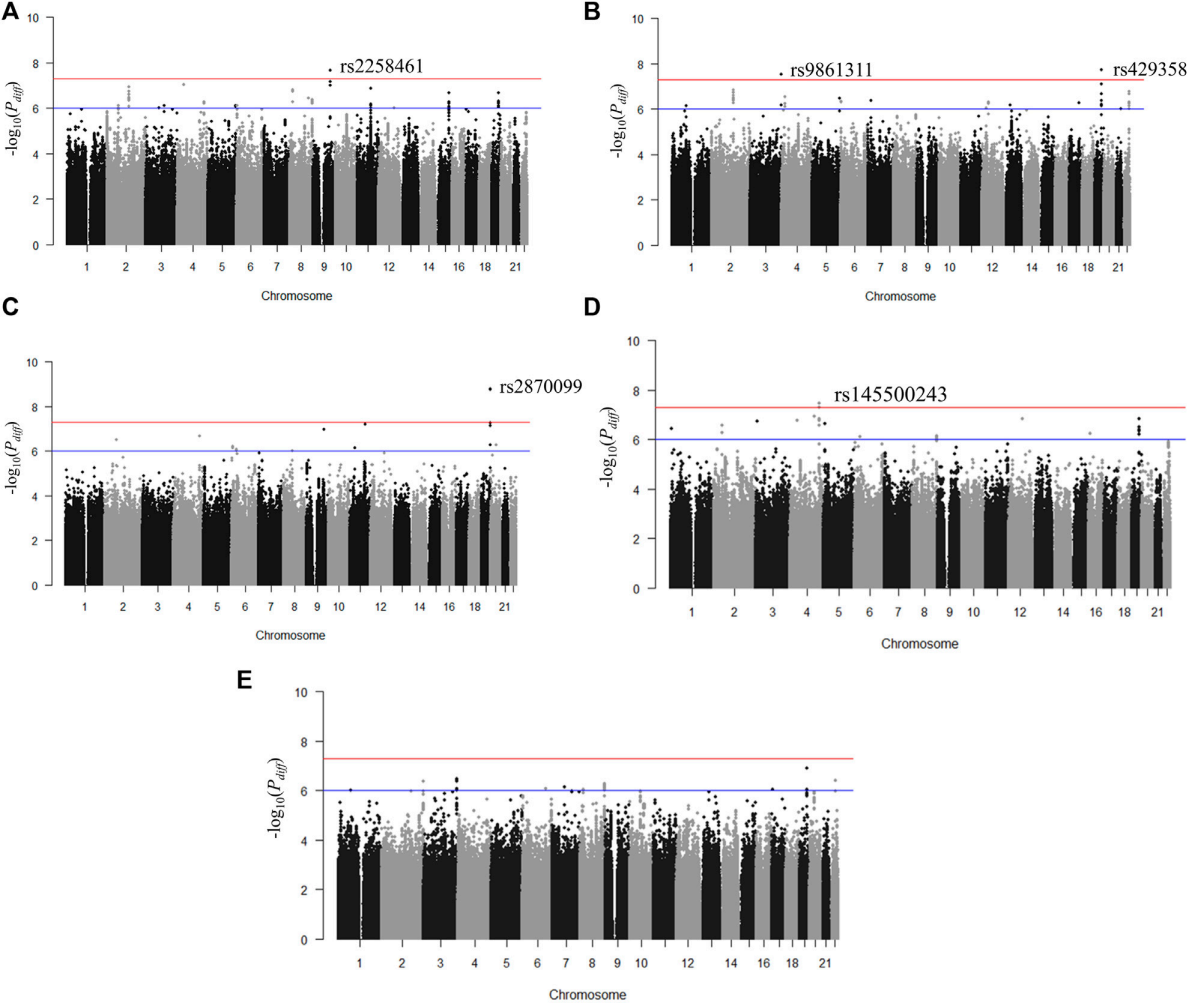
Manhattan plots for *p*-value of T-scores in stratified analyses between groups Q1 and Q4. **(A)** Body mass index, **(B)** body fat percentage, **(C)** waist-hip ratio, **(D)** waist circumference, and **(E)** hip circumference. The red horizontal line (*p* < 5 × 10^–8^) denotes the threshold for genome-wide significance.

We identified genetic variants with different effects between the quartile groups stratified by age, as the study design shown in Figure 1. First, we performed analysis of GWAS in each group of Q1, Q2, Q3, and Q4 separately for each obesity-related phenotype. The association results for each phenotype are shown as Manhattan plots in Supplementary Figure S2, and quantile-quantile plots of results from the analysis of GWAS are shown in Supplementary Figure S3. To select the pair of age groups for performing stratified analysis, we carried out correlation analyses for each pair of GWAS results. For all obesity-related phenotypes, the lowest correlation coefficients were observed in the Q1 and Q4 pair (Supplementary Figure S4). We then performed stratified analyses of GWAS results between the youngest group Q1 and the oldest group Q4, and the differential *p-*value (*P*_*diff*_) for each SNP between Q1 and Q4 was examined using *t*-statistic, as described in the Methods section. We selected genome-wide significant lead SNPs from the results of stratified analyses using the FUMA program (Watanabe et al., 2017). Five lead SNPs satisfied the genome-wide significance level (*P*_*diff*_ < 5 × 10^–8^): one for BMI (rs2258461), two for BFP (rs9861311 and rs429358), one for WHR (rs2870099), and one for WC (rs145500243) (Figure 2 and Table 2). Regional plots of the five lead SNPs were generated using LocusZoom software (Supplementary Figure S5), and suggestive SNPs (*P*_*diff*_ < 1 × 10^–6^) are shown in Supplementary Figure S2.

**TABLE 2:** The results of the age-stratified analysis on obesity-related phenotypes (*P*_*diff*_ < 5 × 10^–8^).

Phenotype	Chromosome	Position^a^	SNP	Nearest gene	Allele^b^ (A1/A2)	*P*_*diff*_
BMI	9	110,323,540	rs2258461	*KLF4*	G/A	2.15E-08
BFP	3	195,760,948	rs9861311	TFRC*	G/C	3.02E-08
	19	4,5,411,941	rs429358	APOE*	C/T	1.91E-08
WHR	19	57,209,395	rs2870099	*AC006115.1*	C/T	1.70E-09
WC	4	168,845,945	rs145500243	*RP11-310I9.1*	T/C	3.46E-08

a Chromosomal positions are based on the 1000 Genomes Project’s haplotype phase 1 in NCBI, build 37 (hg19).

b A1/A2 is minor/major allele of the variant. * denotes the gene within which the lead SNP, locates.

The three SNPs rs9861311, rs429358, and rs145500243 showed a gradual change in effect size along with the age of the quartile groups (Table 3,Supplementary Table S3; Supplementary Figure S6). Minor allele (G allele) of rs9861311 was associated with a decrease in BFP in the youngest age group, but increased BFP with increasing age, and beta coefficients of the association analyses were as follows; Q1: −0.147, Q2: 0.006, Q3: 0.022, and Q4: 0.075. As shown in Supplementary Table S3, mean BFPs in the youngest group were 29.82% in CC genotype subjects and 29.68% in CG + GG genotype subjects, showing 0.14% lower in the G allele carrier. And mean BFPs in the oldest group were 31.96% in CC and 32.16% in CG + GG, showing 0.20% higher in the G allele carrier. That is, if a person has a minor allele of rs9861311, he/she can have lower BFP when he/she is young and higher BFP when older, compared to people with the major allele. rs145500243 was associated with a decrease in WC in the youngest age group, but was associated with an increase in WC in the oldest age group, and beta values were as follows; Q1: −0.596, Q2: −0.419, Q3: −0.209, and Q4: 0.539. Mean WCs in the youngest group were 88.15 cm in CC genotype subjects and 87.57 cm in CT + TT genotype subjects, showing 0.58 cm smaller in the T allele carrier. And those in the oldest group were 91.98 cm and 92.58 cm in CC and CG + GG, respectively, showing 0.60 cm larger in the T allele carrier. Both rs9861311 and rs145500243 continuously changed from a negative to positive effect size from Q1 to Q4, although the statistical significance of these effect sizes did not meet the threshold of *p* < 0.05 in some quartile groups, as shown in Table 3. Minor allele (C allele) of rs429358 was associated with a decrease in BFP in all age groups. The beta values of rs429358 gradually changed to a stronger negative effect as the age of the group increased as follows: −0.021 in Q1, −0.086 in Q2, −0.198 in Q3, and −0.325 in Q4. The mean BFPs in the youngest group were 29.73% in TT genotype subjects and 29.71% in TC + CC genotype subjects, showing 0.02% lower in the C allele carrier. And mean BFPs in the oldest group were 32.21% in TT and 31.81% in TC + CC, showing 0.40% lower in the C allele carrier. This suggest that people with a minor allele of rs429358 may not differ in BFP at a young age compared to people with the major allele, but the difference may gradually increase with age.

**TABLE 3:** The results of GWASs in each age group.

SNP	Phenotypes	Groups	Beta (SE)	*P*
rs2258461	BMI	Q1	−0.076 (0.024)	1.56E-03
		Q2	0.012 (0.024)	6.01E-01
		Q3	−0.001 (0.023)	9.78E-01
		Q4	0.091 (0.022)	3.68E-05
rs9861311	BFP	Q1	−0.147 (0.032)	4.20E-06
		Q2	0.006 (0.030)	8.33E-01
		Q3	0.022 (0.030)	4.67E-01
		Q4	0.075 (0.030)	1.18E-02
rs429358	BFP	Q1	−0.021 (0.043)	6.34E-01
		Q2	−0.086 (0.041)	3.58E-02
		Q3	−0.198 (0.041)	1.59E-06
		Q4	−0.325 (0.040)	1.01E-15
rs2870099	WHR	Q1	0.0018 (0.0004)	5.36E-05
		Q2	0.0002 (0.0004)	7.11E-01
		Q3	0.0010 (0.0004)	2.62E-02
		Q4	−0.0018 (0.0004)	7.57E-05
rs145500243	WC	Q1	−0.596 (0.160)	1.94E-04
		Q2	−0.419 (0.158)	7.91E-03
		Q3	−0.209 (0.159)	1.90E-01
		Q4	0.539 (0.154)	4.64E-04

The data are presented as the Beta (SE). The associations between genetic variants and each obesity-related trait were analyzed using a linear regression model in each age-stratified group, adjusted for age, sex, genotyping array, and PC1∼10. SE, standard error; BMI, body mass index; BFP, body fat percentage; WHR, waist-hip ratio; WC, waist circumference.

Moreover, the five SNPs showed similar age-dependent pattern of effects on the other phenotypes (Supplementary Table S4). For all phenotypes, the effect sizes (beta coefficients) of rs145500243 were the most negative in the youngest group, showing that all obesity-related phenotypes in the minor allele carrier were the lowest among age groups. The effect sizes became reduced in the middle groups of age, Q2 and Q3, and finally became positive in the oldest group, indicating that all obesity-related phenotypes in the minor allele carrier became increased. The rs9861311 polymorphism also showed a continuous change from a negative effect size to a positive effect size with respect to age in four other phenotypes as in BFP, except in HC. Again, rs429358 gradually changed into stronger negative effect sizes as the age of the group increased in four other phenotypes, as in BFP.

### Functional annotation of five lead single nucleotide polymorphisms

For the functional annotation of the five lead SNPs, we examined data from HaploReg V4.1 (Ward and Kellis, 2012), GTEx V8 (Consortium, 2020), GWAS Catalog (Buniello et al., 2019), and PhenoScanner V2 (Kamat et al., 2019) databases. rs9861311 (located in *TFRC*) is an intronic SNP, rs429358 (located in *APOE*) is a missense SNP, and rs2258461 is located 71 kb from 5′ of *KLF4* (Supplementary Table S5). Based on the PhenoScanner data, only rs429358, among the five lead SNPs, has previous reports of genome-wide significant associations and shows associations with diverse age-related diseases, such as Alzheimer’s disease, age-related macular degeneration, coronary artery disease, and cognitive aging, in addition to diverse obesity-related phenotypes (Lambert et al., 2013; Fritsche et al., 2016; Raj et al., 2017; van der Harst and Verweij, 2018) (Supplementary Table S6). Similarly, only rs429358 was reported to be associated with late-onset Alzheimer’s disease, parental lifespan, cognitive aging, and type 2 diabetes, in addition to obesity-related traits, based on the GWAS Catalog (Joshi et al., 2017; Mahajan et al., 2018; Kamboh et al., 2019; Lo et al., 2019) (Supplementary Table S7). Finally, we investigated eQTL data for the five SNPs based on GTEx data (Consortium, 2020) and found eQTL genes for rs9861311 and rs429358. The eQTL genes for rs9861311 were *MUC4* in the skin, thyroid, and liver, and *MUC20* in the adipose tissue, and eQTL gene for rs429358 was *APOC1* in the esophagus and adrenal gland (Supplementary Table S8). There were no functional analysis results for rs2870099 and rs145500243, even with proxy SNPs (*r* ^*2*^ > 0.1).

## Discussion

In this study, we performed age-stratified analyses for five obesity-related phenotypes using 355,335 European from the UKB. To identify genetic variants with differential effects between age groups, we divided samples into quartile groups and compared the association results between the youngest and the oldest groups. We identified five SNPs with significantly different genetic effects in the two age groups. Of these, the direction of the genetic effects for each phenotype was reversed with increasing age in four SNPs: rs2258461 (BMI), rs9861311 (BFP), rs2870099 (WHR), and rs145500243 (WC). For example, the minor allele of rs9861311 lowered mean BFP by 0.14% in the youngest group but increased it by 0.20% in the oldest group. In contrast, the genetic effect of rs429358 on BFP became increased with age. The minor allele of rs429358 lowered mean BFP by 0.02% in the youngest group and lowered it by 0.40% in the oldest group.

The effect of genetic variants on obesity can vary with age (Winkler et al., 2015; Ge et al., 2017; Robinson et al., 2017; Wang et al., 2019). Winkler et al. investigated genetic variants with age-dependent genetic effects on BMI and WHR using 320,485 individuals from GIANT and reported 15 SNPs with statistical significance only on BMI (Winkler et al., 2015). Winkler’s study and ours are similar to each other that these two studies carried out the same method called the stratified analysis. However, there were several differences between two studies as follows. First, Winkler et al. divided samples into two groups based on the age of 50 years, whereas we divided samples into quartile groups and selected the youngest (Q1, 40–50 years) and the oldest (Q4, 64–69 years) groups. Second, Winkler et al. used FDR 5% for multiple testing threshold, whereas we used genome-wide significant *p*-value (5 × 10^–8^) for multiple testing threshold. If we applied a genome-wide significant *P* threshold to Winkler’s results, there would be no statistically significant loci in BMI as well as WHR. Despite these differences, we found that one genetic variant among five significant SNPs in our study showed similar trend in the association results between younger and older groups. rs429358 of our study and rs4420638 of Winkler’s study having a high LD each other (*r* ^*2*^ = 0.69) had significant genetic effects only in old age group. Additionally, we compared 15 SNPs from Winkler’s results with our results on BMI (Supplementary Table S9). Among these 15 loci, 3 SNPs of rs9936385 (*FTO*), rs2867125 (near *TEME18*), and rs4420638 (near *APOC1*) were replicated in our results based on the Bonferroni corrected *P*-threshold, 3.33 × 10^–3^ (=0.05/15).

One of the five lead SNPs, rs9861311, is an intronic SNP located in the *TFRC* gene that encodes the transferrin receptor necessary for cellular iron uptake by receptor-mediated endocytosis. Transferrin is a major iron carrier in blood that maintains cellular iron homeostasis. The sentinel SNP rs9861311 does not show *TFRC* as an eQTL gene in the GTEx data but shows *MUC4* (skin, thyroid, and liver) and *MUC20* (adipose tissue) (Supplementary Table S8). However, several proxy SNPs, including rs34906439 (*r* ^*2*^ = 0.28), rs41298087 (*r* ^*2*^ = 0.28), rs9859260 (*r* ^*2*^ = 0.28), rs2300775 (*r* ^*2*^ = 0.28), and rs3804139 (*r* ^*2*^ = 0.28), showed *TFRC* as the eQTL gene in the spleen, lungs, and skin (Supplementary Table S8). Recently, several publications have supported the association of serum iron and transferrin with lipolysis of adipocytes. Romero et al. examined the role of iron in regulating the energy balance and found that a short course of dietary iron caused a negative energy balance, resulting in a severe wasting phenotype, indicating iron-mediated lipolysis (Romero et al., 2022). Another study showed that TFRC-controlled transferrin contributed to lipolytic effects in isolated rat adipocytes, resulting in a maximal 50% increase in basal lipolysis (Rumberger et al., 2004). In addition, iron participates in key processes, such as oxygen transport, oxidative metabolism, and DNA synthesis and repair related to aging, and elevated iron levels in an organism may have a toxic effect due to its high redox reactivity (Gurzau et al., 2003; Donovan et al., 2006; Theil and Goss, 2009). The cumulative increase in oxidative damage and lowering of the antioxidant defense capacity of the organism are believed to be the main features of aging (Beckman and Ames, 1998; Liguori et al., 2018). Supporting the idea that iron homeostasis may be involved in the process of age-related disease, a study demonstrated that transferrin concentration in circulation was related to the development of age-related macular degeneration disease (Wysokinski et al., 2013). Based on these studies, we hypothesized that rs9861311 affects the expression of *TFRC* that changes the serum iron concentration or the cellular iron concentration, leading to lipolysis of adipose tissue, which may be linked to age *via* oxidative damage and antioxidant defense capacity. This hypothesis awaits further investigation.

The genetic variant rs429358 is a missense variant, replacing a cysteine with arginine at amino acid 112 (Cys112Arg) of *APOE* gene. Among the five lead SNPs, rs429358 was the only SNP that was reported significant associations with various traits in previous GWASs. As shown in Supplementary Tables S6,S7, the traits associated with rs429358 include diverse age-related diseases, such as Alzheimer’s disease, dementia, coronary artery disease, age-related macular degeneration, and cognitive decline (Lambert et al., 2013; Fritsche et al., 2016; Raj et al., 2017; van der Harst and Verweij, 2018; Rongve et al., 2019). In addition, it is also associated with lifespan and longevity (Timmers et al., 2020). APOE binds to lipoprotein particles and transports lipids within the particles through circulation (Zannis et al., 2004). By binding to the LDL receptor, APOE mediates lipid uptake into cells and plays a crucial role in lipid metabolism and homeostasis. Furthermore, the new roles of APOE have been reported to be a multifunctional protein in immune cells and the brain, such as the inflammation and aggregation of amyloid beta, respectively (Tudorache et al., 2017).

Three APOE isoforms, ε2, ε3, and ε4, are formed by a combination of two missense variants (rs429358 and rs7412 in *APOE*), and the ε3 allele is the most common (∼78% globally), followed by ε4 (∼14%) and ε2 (∼8%) (Zannis and Breslow, 1981; Weisgraber et al., 1982; Egert et al., 2012; Husain et al., 2021). Among these isoforms, the ε4 isoform formed by carrying the C allele of rs429358 and C allele of rs7412 is well known for its association with a 3–4 fold increased incidence of Alzheimer’s disease as with ε4 heterozygote and 9–15 fold with ε4 homozygotes compared to those in non-carriers of ε4 (Farrer et al., 1997; Neu et al., 2017). The ε4 allele or rs429358 has also been previously reported to be associated with BMI and WC decreasing obesity with the C allele of rs429358, which is similar to the results of this study (ε2 > ε3 > ε4 for BMI and WC) (Tejedor et al., 2014; Palmer et al., 2021). In addition, Kulminski et al. (2019) reported that the association of rs429358 minor allele (C allele) with decreased in BMI only in old subjects of the study. Similarly, we demonstrated that the negative effect of rs429358 became stronger as the group age increased for all five obesity-related phenotypes (Supplementary Table S4). Also, rs429358 was also reported to be associated with serum cholesterol and triglyceride levels (Tejedor et al., 2014; Palmer et al., 2021). We also found an association between rs429358 and HDL and LDL, as shown in Supplementary Table S10, confirming previous results.

Another SNP for the formation of the APOE isoform, rs7412, is also well known for its effect on plasma lipid levels and obesity-related phenotypes, similar to rs429358 (Tejedor et al., 2014). However, the association of rs7412 with obesity-related phenotypes showed the opposite trend. rs7412 increases BMI and WC, unlike rs429358 (Tejedor et al., 2014). We examined whether the effect of rs7412 is also age-dependent, but we did not find a clear age-dependency (Supplementary Table S11). Although the effects of rs7412 on BMI, BFP, and HC in Q2 and Q3 were stronger than those in Q1, the effects in Q4 were weaker than those in Q2 and Q3. Moreover, the *P*_*diff*_ of rs7412 was not significant for any obesity-related phenotype when the difference between the Q1 and Q4 groups was tested.

The mechanism for the association of APOE with obesity and body fat mass is speculated to be attributed to its role in regulating the expandability and functionality of adipose tissues (Huang et al., 2006; Arbones-Mainar et al., 2008; Huang et al., 2009a; Huang et al., 2009b; Li and Liu, 2014). However, we could not explain why the genetic effects of rs429358 changed with age. This may be related to the fact that rs429358 is strongly associated with lifespan. Timmers et al. (2020) investigated genetic variants related to human aging using a multivariate meta-analysis of parental lifespan, health span, and longevity in UKB participants. They found rs429358 as the most significant multivariate SNP, and the average increase in parental lifespan was 12.7 months per T allele of rs429358. Notably, the allelic effect of rs429358 (T allele) on lifespan increased as the sample age increased, increasing the effect by 32% for every 10 years increase in parental survival.

rs2258461 is an intergenic SNP with *KLF4* as the nearest gene, which encodes a protein that belongs to the Kruppel family of transcription factors. Based on the GWAS catalog, *KFL4* is associated with breast and prostate cancers, but no other phenotypes are associated with genome-wide significance levels. Several members of the KLF gene family affect lipid and glucose metabolism as well as adipocyte differentiation, thus influencing energy homeostasis and contributing to obesity (Pollak et al., 2018). Mice with myeloid-specific *KLF4* deletions tended to express diet-induced obesity, glucose intolerance, and insulin resistance (Liao et al., 2011). In addition, the expression of *KLF4* in vascular endothelium decreases with age (Hsieh et al., 2017a; Hsieh et al., 2017b). We speculate based on these results that the role of KLF4 in obesity may change with age. Therefore, we hypothesize that the effect of rs2258461 on the expression of *KLF4* may be modified by age, decreasing the level of KLF4 in old subjects resulting in increased BMI. This hypothesis awaits further study.

To ascertain further functional information of rs2870099 and rs145500243, we tested SNPs within 1 Mb flanking these two SNPs. As a result, we found GWAS signals relevant to obesity around rs2870099 as follows: rs34863160 (*ZNF470*), rs11670527 (*DUXA*, *ZNF264*), and rs16987303 (*ZNF471*) were associated with birth weight, BMI, and height, respectively. We also found GWAS signals around rs145500243 as follows: rs13133687 (*ANXA10*), rs538044222 (*DDX60L*, *DDX60*), and rs1963569 (*DDX60L*) were associated with Alzheimer disease (age at onset), BMI-adjusted HC, and energy intake, respectively.

Our study had several limitations. Owing to the UKB sample characteristics, subjects in this study were aged 40–69 years within a narrow age range, which made it challenging to find genetic variants that differentially affect obesity-related phenotypes based on age. Therefore, further studies using cohorts with a wider age range, including young subjects, are needed to identify additional genetic variants with differential effects based on age. Another limitation is that our findings in European samples were not validated in other ethnic populations, and replication studies using other ethnic populations are needed for these five lead SNPs.

In summary, we performed age-stratified analysis of GWAS for obesity-related phenotypes (BMI, BFP, WHR, WC, and HC) using European participants in the UKB and identified five lead SNPs that differed according to age. In particular, the C allele of rs429358 in *APOE* gradually decreases BFP as one grows older in the range of 40–69 years. Our findings may increase the understanding of the underlying mechanisms by which genetic variants differentially influence obesity-related phenotypes based on age, which could provide the better target biomarker for the age-dependent treatment of obesity. Also, the identification of age-affected genetic variants underscores the importance of age in precision medicine using genetic variants, and allows to construct age-specific genetic risk scores for more precise disease prediction based on age.

## Data Availability

The original contributions presented in the study are included in the article/Supplementary Materials, further inquiries can be directed to the corresponding authors.
