# Supplementary material for: Identification of five genetic variants with differential effects on obesity-related traits based on age
Source: Front Genet. 2022 Oct 7;13:970657. doi: 10.3389/fgene.2022.970657 (PMC9585212; doi:10.3389/fgene.2022.970657)
Supplement: Supplementary file 2 [file Image1.PDF]

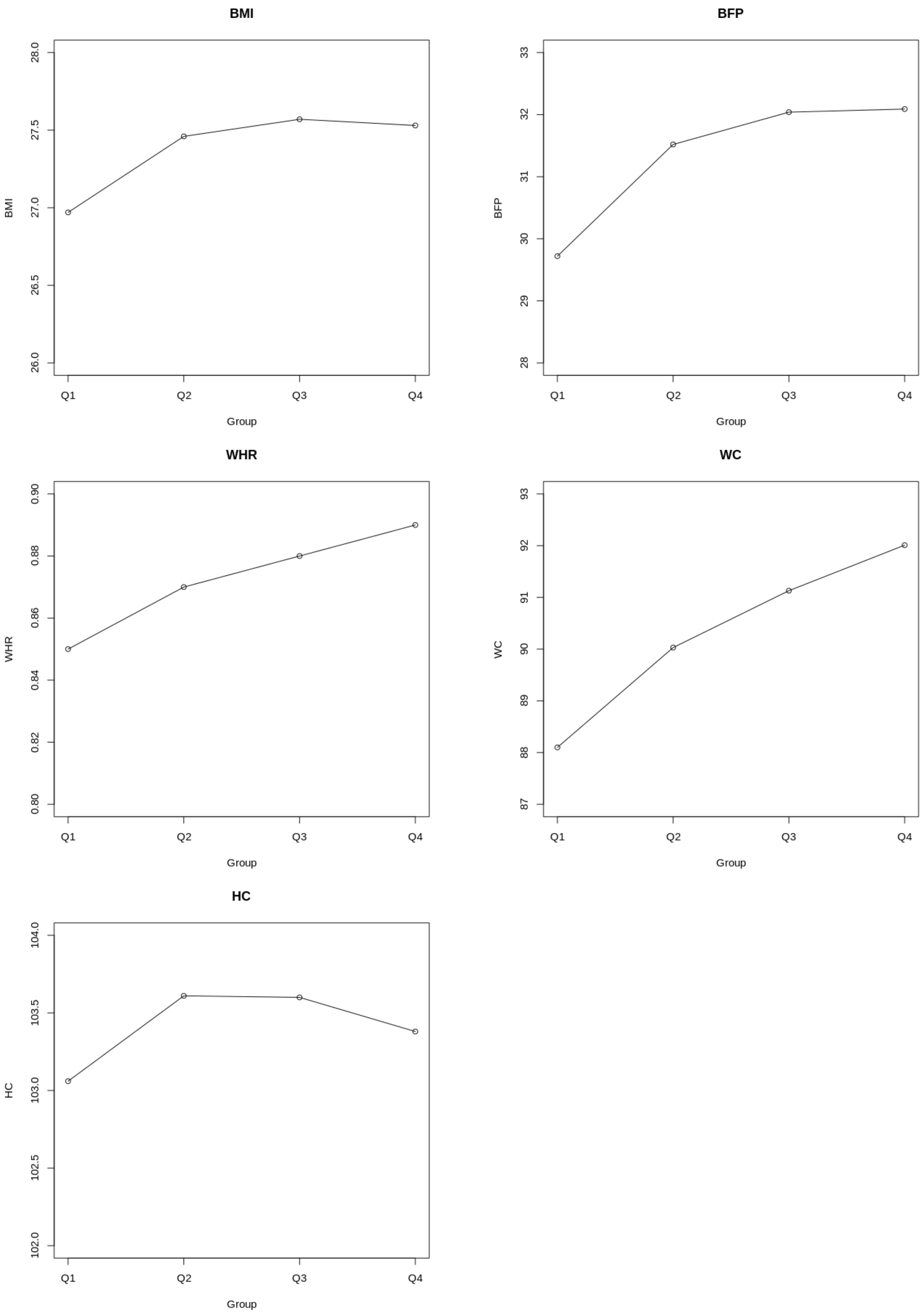

Supplementary Figure S1. Mean values for obesity-related traits such as BMI, BFP, WHR,WC and HC in each group.

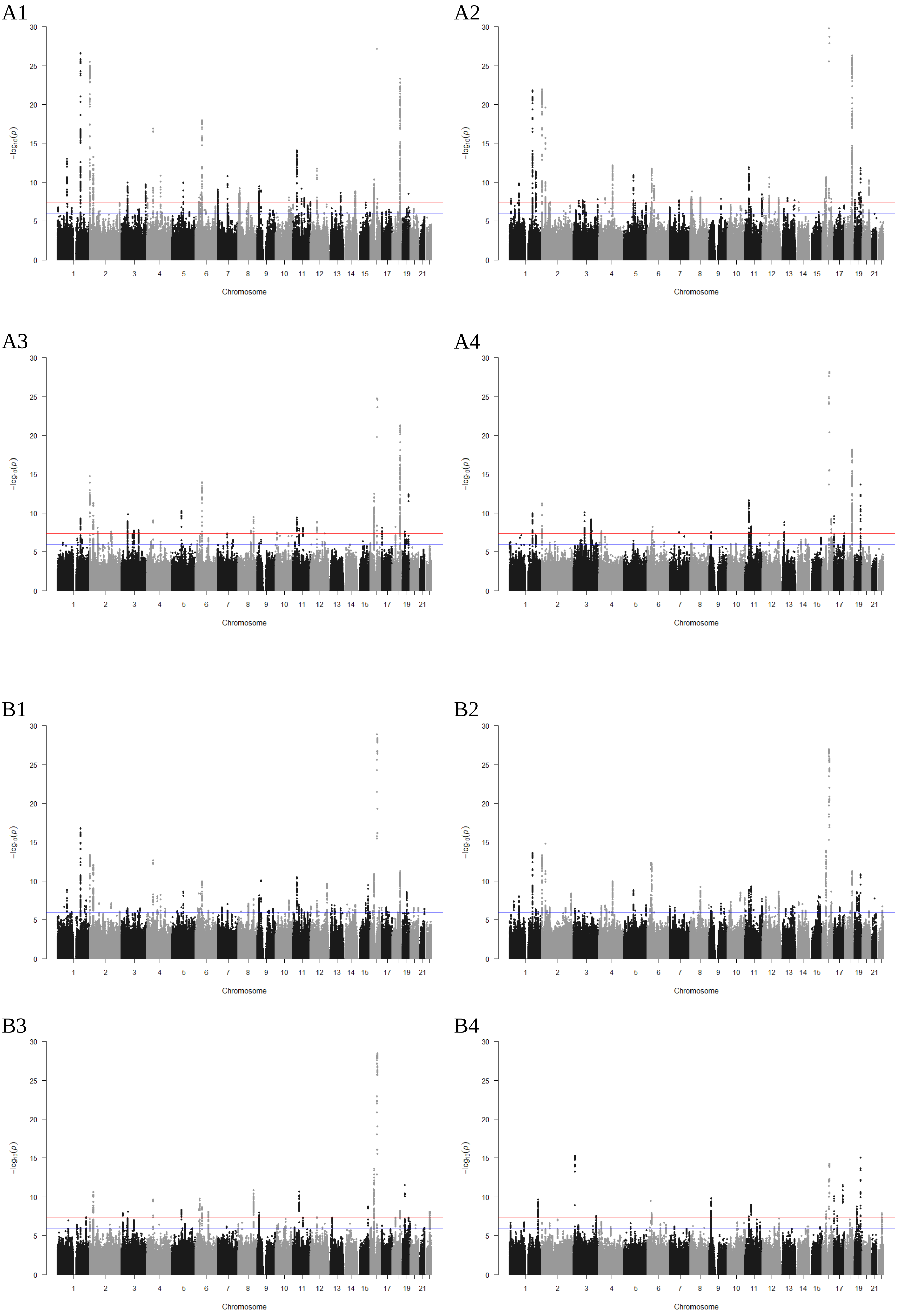
A1
A2
A3
A4
B1
B2
B3
B4
0 5 10 15 20 25 30
$-\log_{10}(p)$
1 2 3 4 5 6 7 8 9 10 11 12 13 14 15 17 19 21
Chromosome

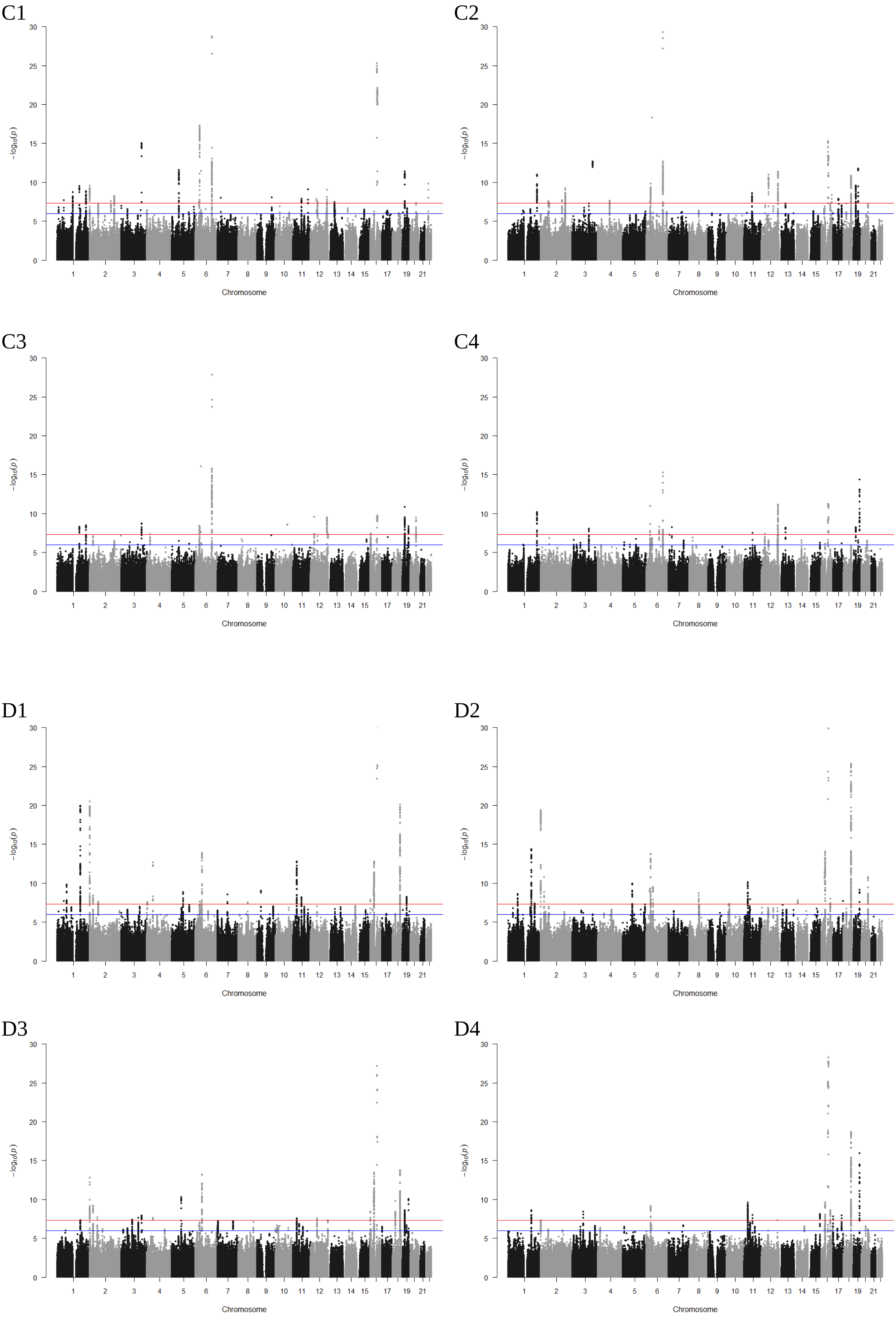
C1
C2
C3
C4
D1
D2
D3
D4
−log10(p)
Chromosome

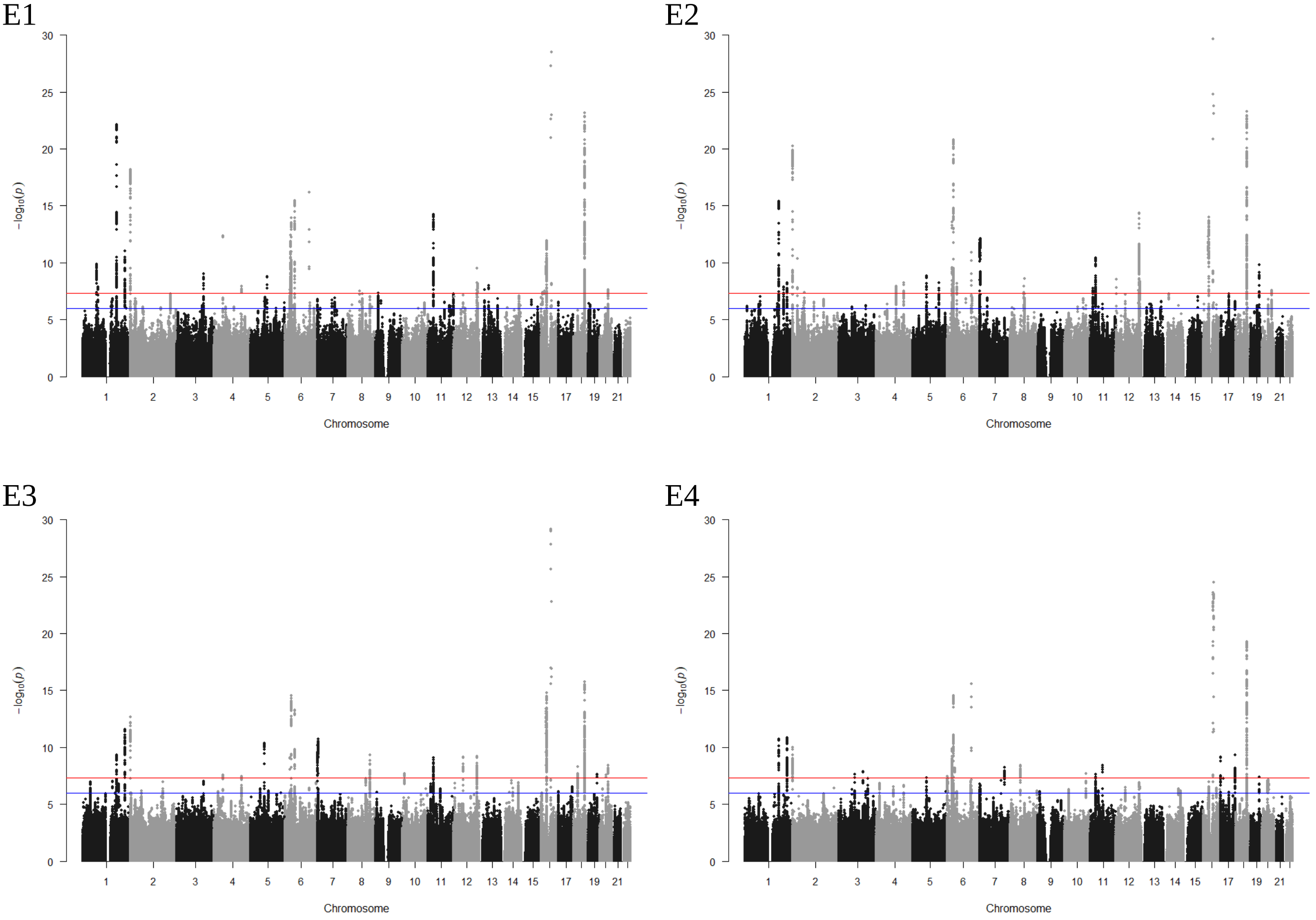

Supplementary Figure S2. Manhattan plots of genome-wide association studies on five obesity-related traits [BMI (A1-A4), BFP (B1-B4), WHR (C1-C4), WC (D1-D4), and HC (E1-E4)] according to quartile groups based on age in UKB. The genome-wide significance level is set at P-value 5×10^{-8} with a red line, and the suggestive level is set at P-value 1×10^{-6} with a blue line.

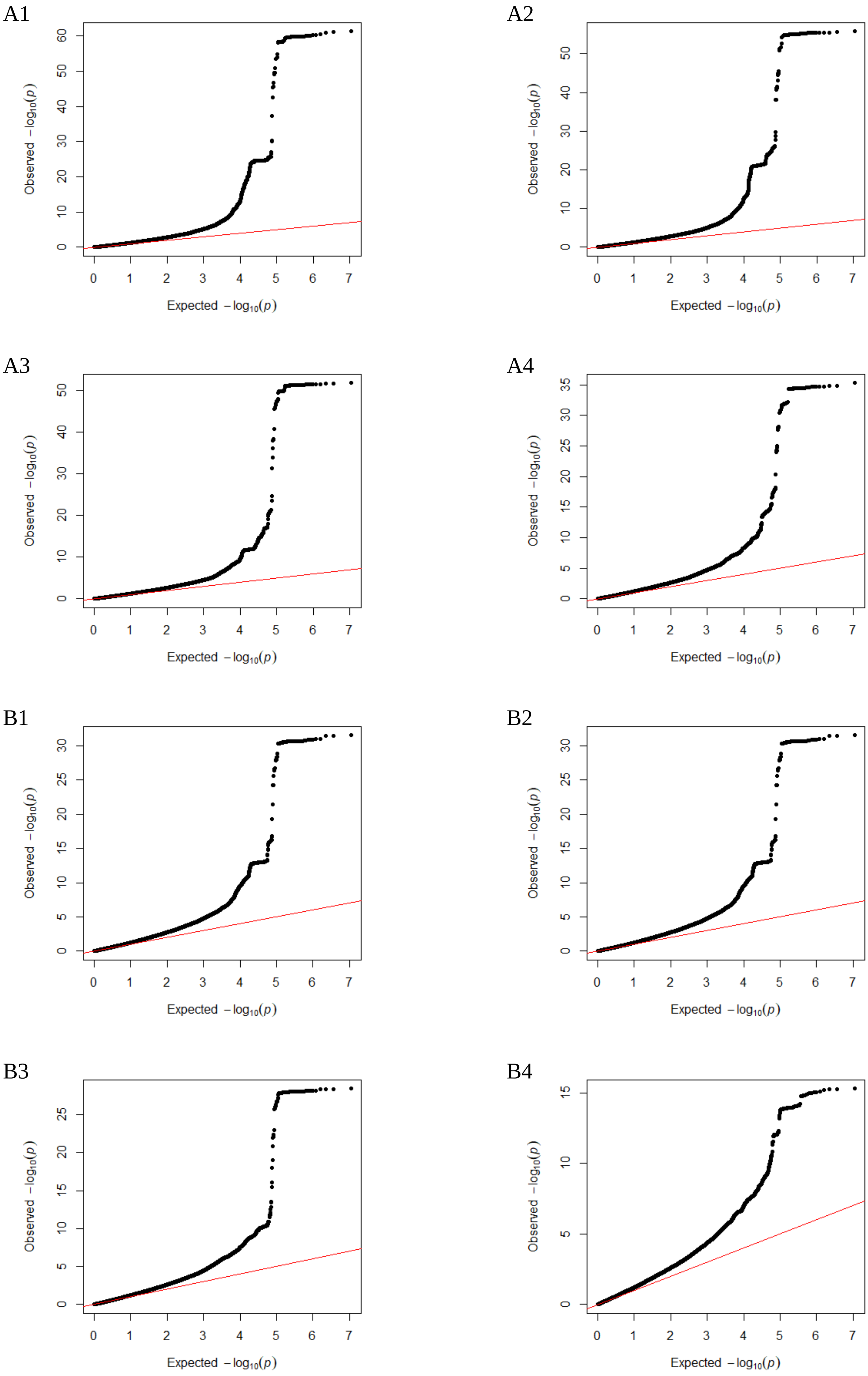

A1
A2
A3
A4
B1
B2
B3
B4
Observed $-\log_{10}(p)$
Expected $-\log_{10}(p)$

C1

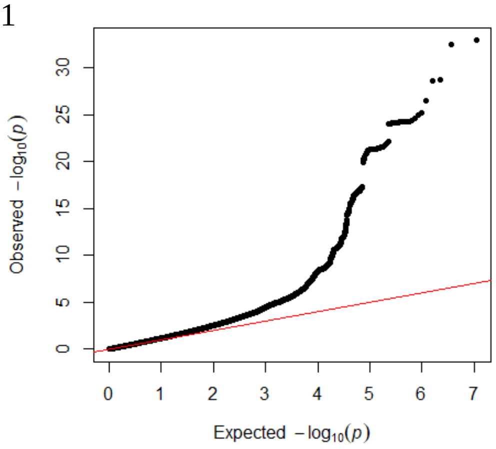

C2

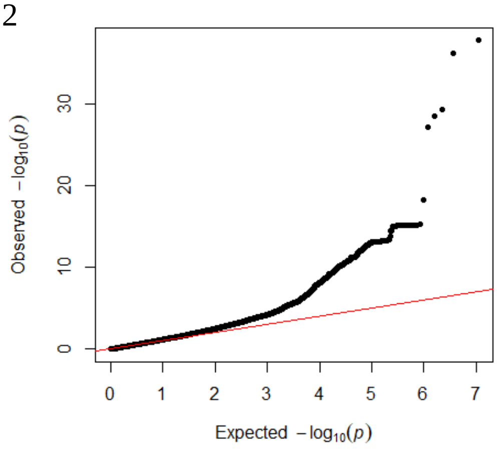

C3

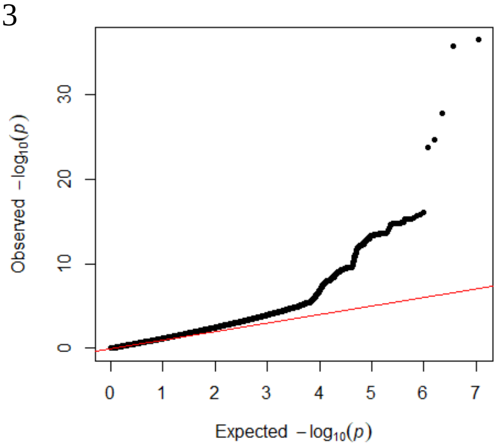

C4

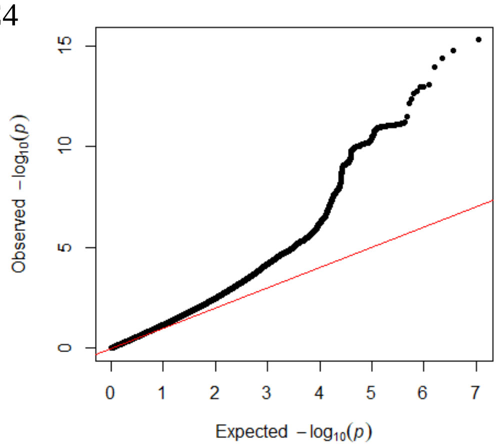

D1

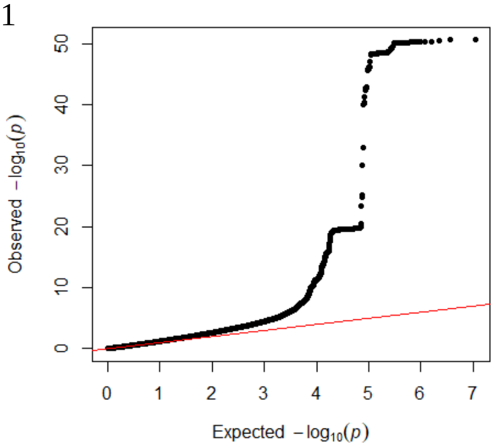

D2

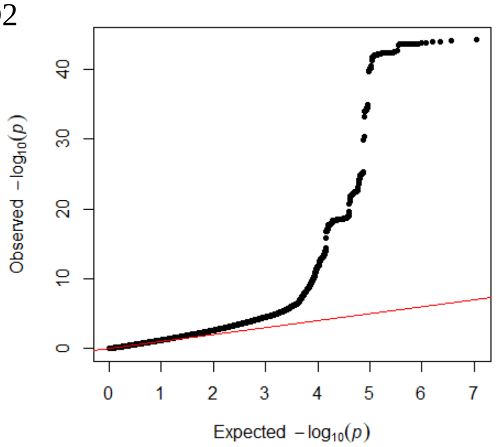

D3

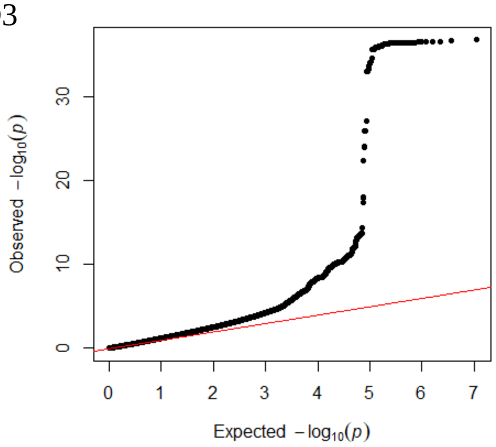

D4

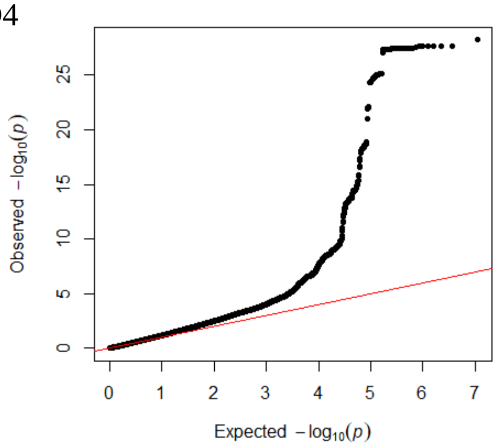

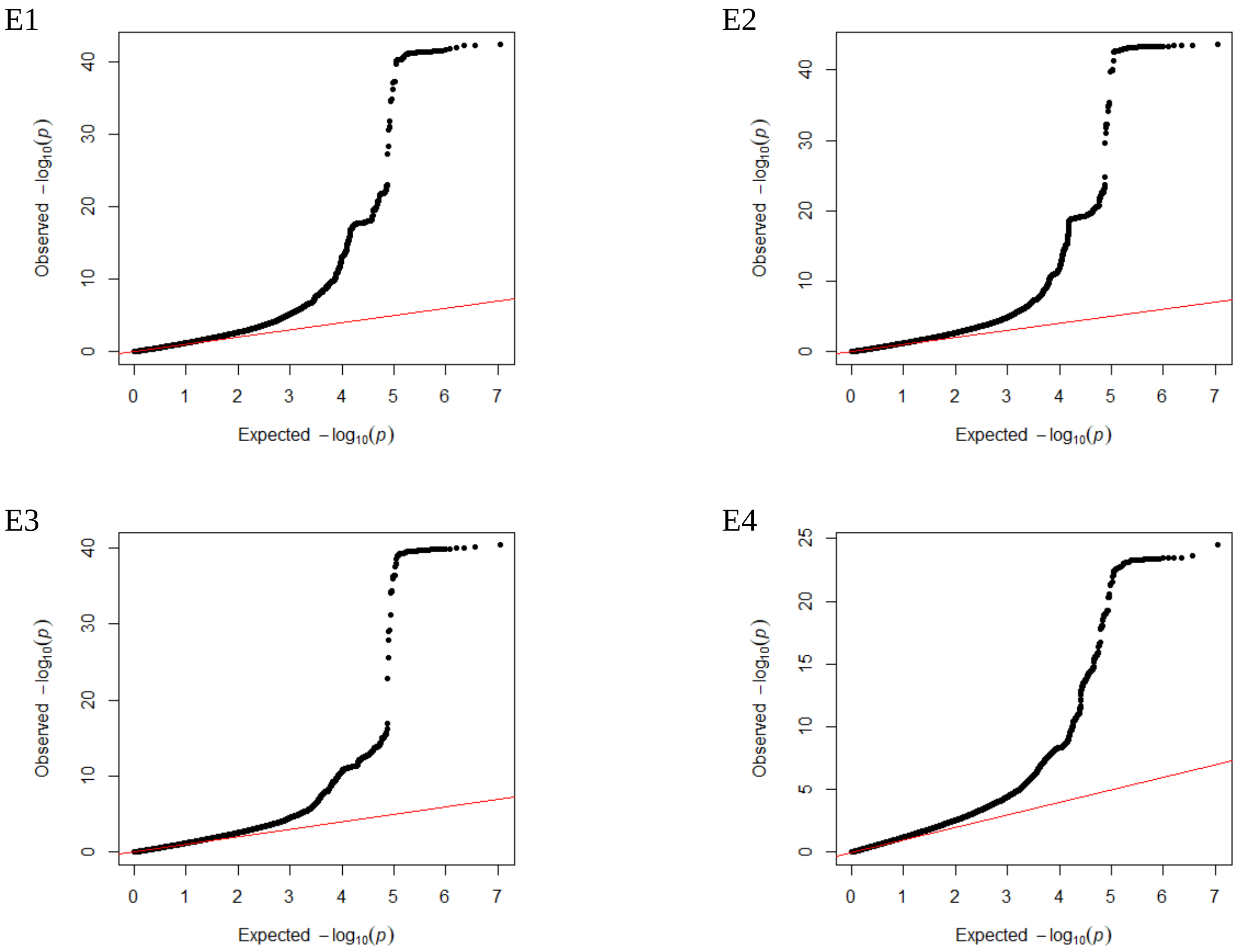

Supplementary Figure S3. Quantile-quantile plots of genome-wide association studies on five obesity-related traits [BMI (A1-A4), BFP (B1-B4), WHR (C1-C4), WC (D1-D4), and HC (E1-E4)] according to quartile groups based on age in UKB.

A. BMI

	Q1	Q2	Q3	Q4
**Q1**	1			
**Q2**	0.188	1		
**Q3**	0.171	0.176	1	
**Q4**	**0.164**	0.173	0.169	1

B. BFP

	Q1	Q2	Q3	Q4
**Q1**	1			
**Q2**	0.179	1		
**Q3**	0.166	0.168	1	
**Q4**	**0.159**	0.163	0.163	1

C. WHR

	Q1	Q2	Q3	Q4
**Q1**	1			
**Q2**	0.142	1		
**Q3**	0.130	0.133	1	
**Q4**	**0.120**	0.124	0.123	1

D. WC

	Q1	Q2	Q3	Q4
**Q1**	1			
**Q2**	0.166	1		
**Q3**	0.149	0.156	1	
**Q4**	**0.141**	0.145	0.146	1

E. HC

	Q1	Q2	Q3	Q4
**Q1**	1			
**Q2**	0.168	1		
**Q3**	0.151	0.156	1	
**Q4**	**0.144**	0.151	0.147	1

Supplementary Figure S4. The correlation matrix for Q1, Q2, Q3, and Q4

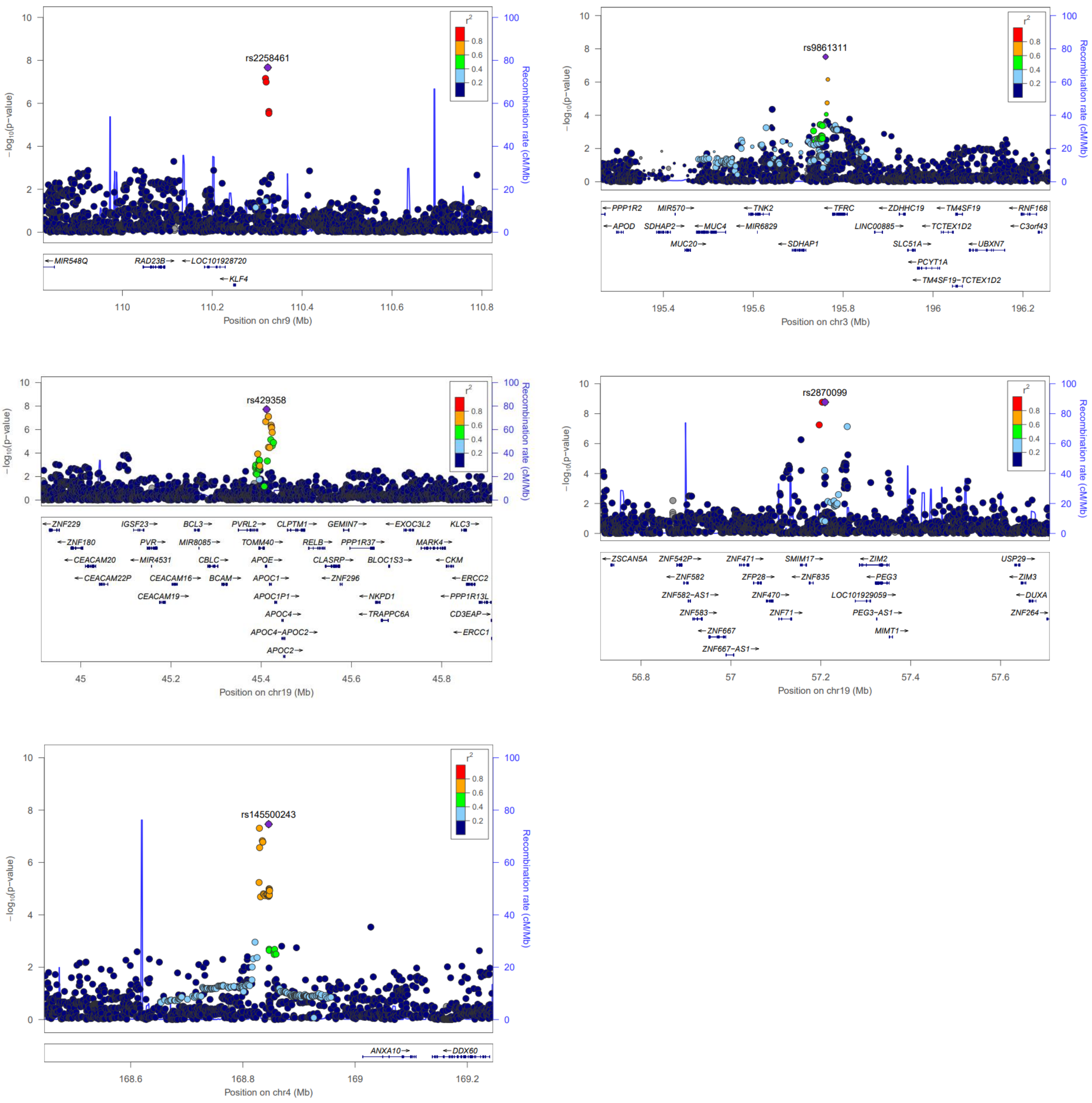

Supplementary Figure S5. Regional plots of the five lead SNPs described by LocusZoom. rs2258461 for BMI, rs9861311 and rs429358 for BFP, rs2870099 for WHR, and rs145500243 for WC are depicted for their plots.

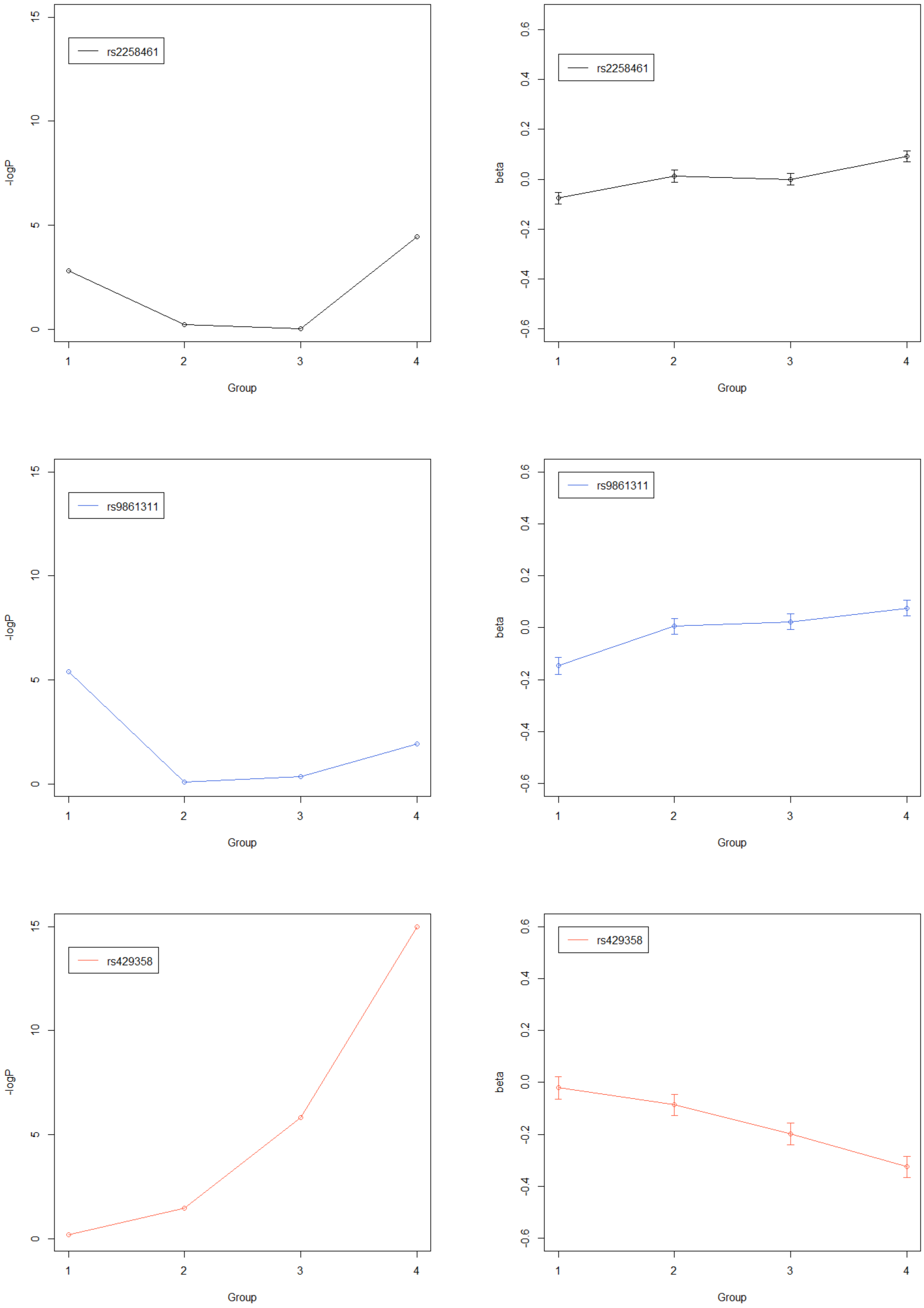
rs2258461
-logP
Group
beta
rs9861311
rs429358
0
5
10
15
-0.6
-0.4
-0.2
0.0
0.2
0.4
0.6
1
2
3
4

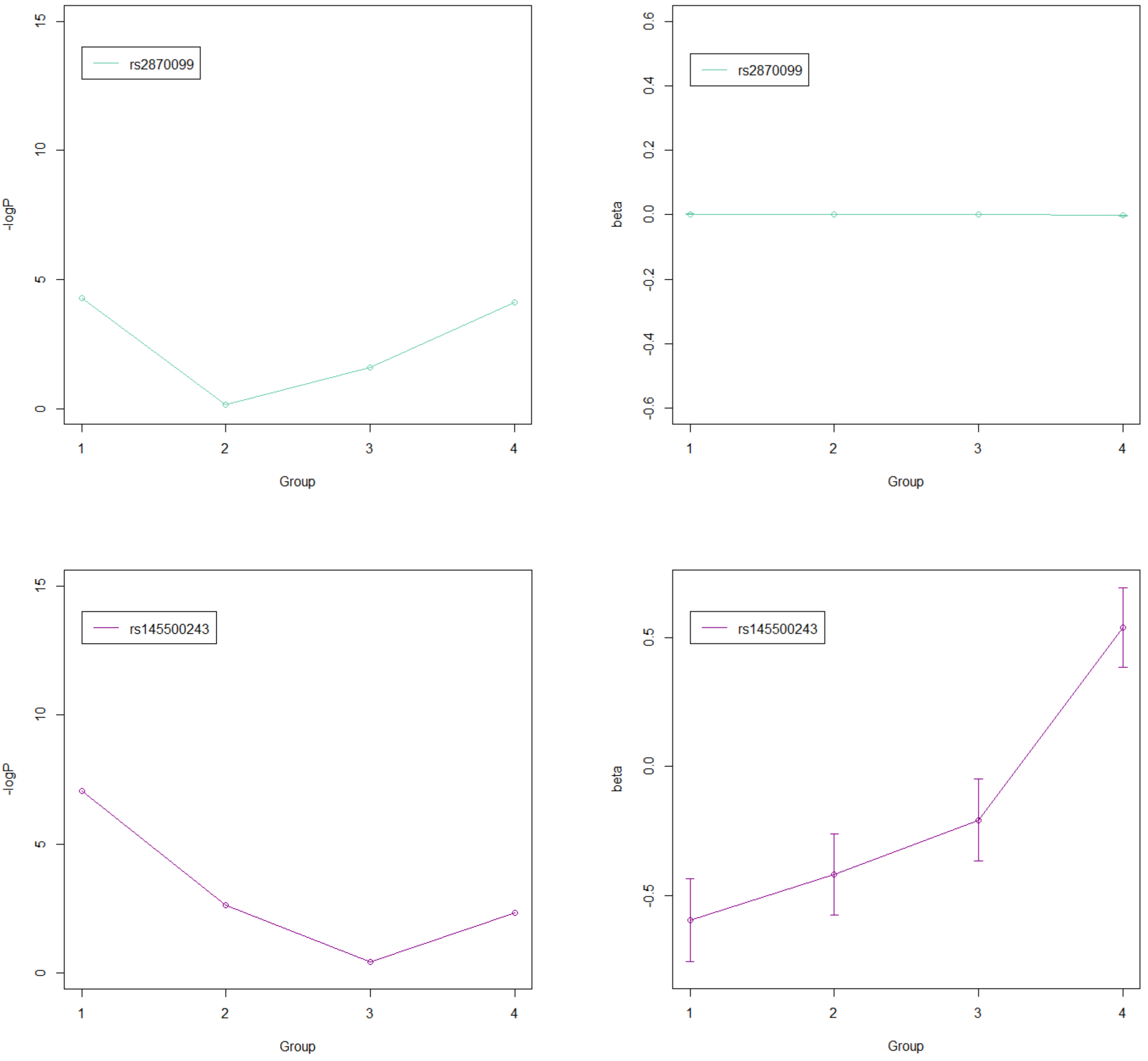

Supplementary Figure S6. Association *P*-values and effect sizes of the five lead SNPs in each quartile group. The association P-value (left) and beta-se (right) as y-axis are shown as 1 (Q1 group), 2 (Q2 group), 3 (Q3 group), and 4 (Q4 group) on the x-axis. These graphs show the association of rs22588461 with BMI, rs9861311 and rs429358 with BFP, rs2870099 with WHR, and rs145500243 with WC, in which phenotype the lead SNPs were identified using stratified analysis.
